# Formimidoyltransferase cyclodeaminase prevents the starvation-induced liver hepatomegaly and dysfunction through downregulating mTORC1

**DOI:** 10.1371/journal.pgen.1009980

**Published:** 2021-12-23

**Authors:** Wenfeng Zhang, Chaoying Wu, Rui Ni, Qifen Yang, Lingfei Luo, Jianbo He

**Affiliations:** 1 Institute of Developmental Biology and Regenerative Medicine, Southwest University, Beibei, Chongqing, China; 2 Chongqing Key Laboratory of Oral Diseases and Biomedical Sciences, Yubei, Chongqing, China; University of Michigan, UNITED STATES

## Abstract

The liver is a crucial center in the regulation of energy homeostasis under starvation. Although downregulation of mammalian target of rapamycin complex 1 (mTORC1) has been reported to play pivotal roles in the starvation responses, the underpinning mechanisms in particular upstream factors that downregulate mTORC1 remain largely unknown. To identify genetic variants that cause liver energy disorders during starvation, we conduct a zebrafish forward genetic screen. We identify a *liver hulk (lvh)* mutant with normal liver under feeding, but exhibiting liver hypertrophy under fasting. The hepatomegaly in *lvh* is caused by enlarged hepatocyte size and leads to liver dysfunction as well as limited tolerance to starvation. Positional cloning reveals that *lvh* phenotypes are caused by mutation in the *ftcd* gene, which encodes the formimidoyltransferase cyclodeaminase (FTCD). Further studies show that in response to starvation, the phosphorylated ribosomal S6 protein (p-RS6), a downstream effector of mTORC1, becomes downregulated in the wild-type liver, but remains at high level in *lvh*. Inhibition of mTORC1 by rapamycin rescues the hepatomegaly and liver dysfunction of *lvh*. Thus, we characterize the roles of FTCD in starvation response, which acts as an important upstream factor to downregulate mTORC1, thus preventing liver hypertrophy and dysfunction.

## Author summary

Under starvation, the liver initiates a series of metabolic adaptations to maintain energy homeostasis that is critical for survival. During this process, mTORC1 pathway is downregulated to reduce anabolism and promote catabolism, ensuring adequate usage of limited resources. However, mechanisms underlying the downregulation of mTORC1 remain incompletely understood. In a zebrafish genetic screen aiming to characterize factors important for starvation response in the liver, we identify an *ftcd* mutation that causes liver hypertrophy and dysfunction under fasting. FTCD acts upstream to inactivate mTORC1 in response to starvation. Our work reveals previously unappreciated roles of FTCD in the responses to energy stress through modulating mTORC1 activities, moreover implicates a potential liver disorder risk of FTCD deficiency under the circumstances of starvation.

## Introduction

The liver is a crucial digestive organ that acts as a pivotal hub for many physiological processes [1,2]. During starvation, the liver initiates a series of metabolic adaptations to maintain systemic energy homeostasis, such as reducing hepatocyte volume, supply of glucose to the circulation from hepatic glycogen, promoting gluconeogenesis, and boosting ketogenesis [3–5]. Hepatomegaly is one of the most common and representative signs indicating the disorder of energy homeostasis, which eventually leads to liver failure [6,7].

The mammalian target of rapamycin complex 1 (mTORC1) pathway plays vital roles in regulating energy balance by controlling multiple cellular functions, such as cell size, genetic transcription, protein synthesis, autophagy, and ribosome biogenesis [8–10]. Under fasting, the activation of mTORC1 pathway is inhibited in the liver [11,12], which is implicated in controlling the reduction of hepatocyte size and reducing anabolism and promoting catabolism [10]. Liver-specific DEPTOR (DEP-domain containing mTOR-interacting protein) deletion mice shows sustained mTORC1 activation upon fasting that leads to hepatocellular hypertrophy [13]. Activation of mTORC1 through hepatocyte-specific deletion of the tuberous sclerosis complex 1 (Tsc1) induces liver inflammation and carcinogenesis in aged mice, but not the young mice [14]. In zebrafish, *tsc2* mutants exhibit large hepatocytes, and inhibition of mTORC1 activity can suppress hepatomegaly [15]. Therefore, under the circumstances of starvation, downregulation of mTORC1 activity plays a pivotal role in reducing cell size and maintenance of energy homeostasis in the liver. However, key molecules that inactivate mTORC1 activity in response to starvation remain a mystery.

Formimidoyltransferase cyclodeaminase (FTCD) is a folate-dependent enzyme that can catalyze two reactions in the histidine degradation pathway and channels one-carbon units from formiminoglutamate to the folate pool [16,17]. As an octamer structure, FTCD could bind to the Golgi complex and promote bundling of vimentin filaments, so it performs structural functions in addition to enzymatic functions [18,19]. FTCD, highly expresses in the liver, serves as a liver-specific autoantigen in patients with autoimmune hepatitis [20] and a candidate tumor suppressor gene in hepatocellular carcinoma [21,22]. The *ftcd* mutation causes glutamate formiminotransferase deficiency (FTCD deficiency) which is a common inborn defect in the folate metabolism [23,24]. No previous report has suggested any connection between FTCD and starvation response.

The zebrafish is an ideal vertebrate model to study liver development, regeneration, and diseases [25,26]. The zebrafish forward genetic screen randomly covers the whole genome without bias, which becomes a powerful tool to obtain genetic mutants with unique phenotypes. In this study, to identify key regulatory molecules involved in the starvation response, we carried out a zebrafish N-ethyl-N-nitrosourea (ENU) forward genetic screen for mutants exhibiting hepatomegaly phenotypes under fasting, but with relatively normal liver under feeding. A mutant named *liver hulk* (*lvh*) was identified, in which the *ftcd* gene was mutated. Further studies showed that the mTORC1 sustained hyperactivation in the liver of *lvh* mutant under fasting, which caused hepatomegaly, and ultimately led to liver failure and earlier death. These findings reveal FTCD as a key factor to downregulate mTORC1 activity under the circumstances of fasting, mediating starvation response, and protecting the liver from hepatomegaly.

## Results

### The zebrafish *lvh* mutant exhibits hepatomegaly and enlarged hepatocyte size under fasting

**Fig 1:**
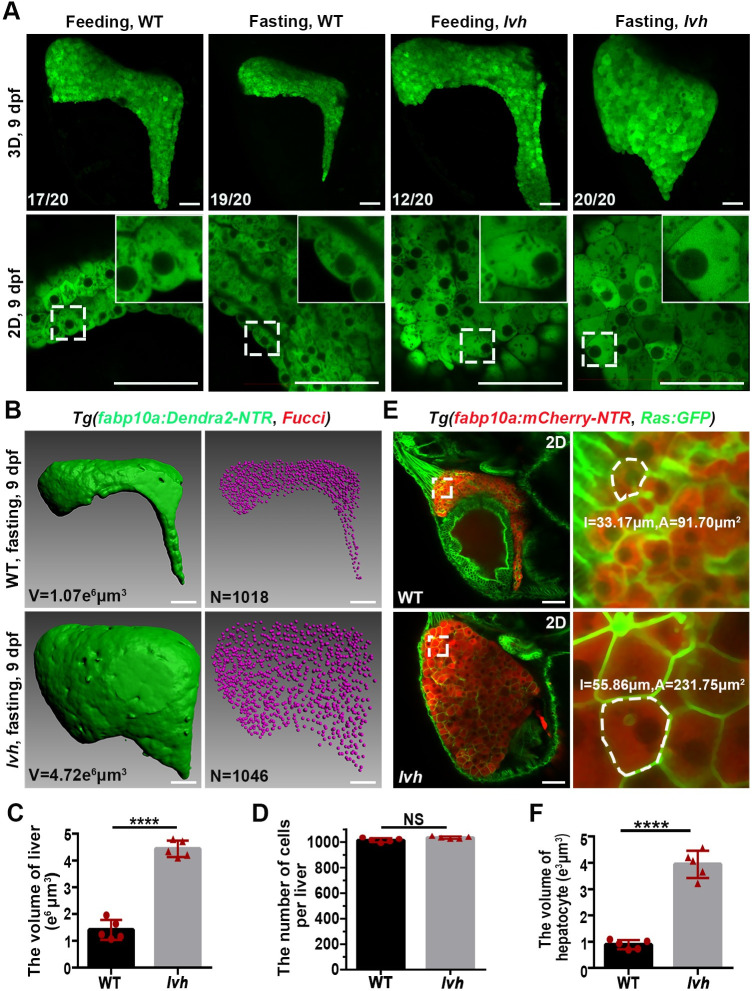
The *lvh* mutant exhibits hepatomegaly and enlarged hepatocyte size under fasting. (A) Confocal 3D projection and 2D single-optical section images of the liver under feeding and fasting in the wild-type or *lvh* mutant at 9 dpf. Higher magnification images showing single hepatocytes are displayed upright. (B) 3D reconstruction images showing the liver volumes and hepatocyte nuclei of the wild-type and *lvh* mutant at 9 dpf. (C) Unpaired Student’s *t*-test for the liver volume of wild-type (n = 5) and *lvh* (n = 5). (D) Unpaired Student’s *t-*test for the number of hepatocytes per liver of wild-type (n = 5) and *lvh* (n = 5). (E) Single-optical section images showing livers of the wild-type and *lvh* mutant at 9 dpf. Higher magnification images of single hepatocytes (dashed frames) are displayed. (F) Unpaired Student’s *t*-test for single hepatocyte volume in the wild-type (n = 5) and *lvh* (n = 5). NS, not significant. ****P<0.0001. WT, wild-type. Data are represented as mean±SD. Scale bars, 50 μm.

To identify factors regulating starvation response, we conducted an ENU forward genetic screen under the transgenic background *Tg(fabp10a*:*Dendra2-NTR)*^*cq1*^, in which hepatocytes were labeled by Dendra2 driven by the hepatocyte-specific promoter liver fatty acid-binding protein 10a (*fabp10a*) [27]. Zebrafish larvae are able to take food, and the liver is fully functional from 5 days post fertilization (dpf) on [28]. Fasting was applied on the larvae from 5 days post fertilization (5 dpf), and the mutants with hepatomegaly phenotype at 9 dpf were screened. A recessive mutant named *liver hulk* (*lvh*^*cq107*^), which exhibited liver hypertrophy under fasting, but maintained relatively normal liver size under feeding, was identified (Fig 1A). The morphology of *lvh* mutant larvae remained normal under fasting circumstances (S1A Fig). Living imaging showed that the enlargement of liver size in *lvh* under fasting was clearly visible at 7 dpf and became more evident at 9 dpf and 11 dpf (S1B Fig).

High-resolution images showed that although the volume of wild-type hepatocytes was reduced in response to fasting, the sizes of *lvh* hepatocytes significantly increased under fasting (Fig 1A). We further validated whether the fasting-induced hepatomegaly in *lvh* was mainly caused by enlarged cell size or increased cell number also contributed. The hepatocyte cell number was quantified using the *Tg(fabp10a*:*Dendra2-NTR; Fucci)* transgenic line that labeled hepatocytes and their nuclei. Although the liver volume of *lvh* was approximately quadruple of that of siblings, the number of hepatocytes showed no significant difference (Fig 1B–1D). Under the *Tg(fabp10a*:*mCherry-NTR; Ras*:*GFP)* transgenic background, in which the cell membrane was labeled with GFP, the volume of a single hepatocyte in the *lvh* mutant exhibited approximately four times as large as siblings (Fig 1E and 1F). These results indicate that the fasting-induced hepatomegaly in *lvh* is caused by enlarged volume of hepatocytes, but not increased cell number.

We then check whether the fasting-induced hypertrophy in *lvh* is specific to the liver or also effective to other digestive organs. Expressions of the intestinal marker fatty acid-binding protein 2 (*fabp2*), the pancreatic islet marker *insulin*, and the exocrine pancreas marker *trypsin* at 8 dpf remained unaffected in *lvh* under fasting (S2A–S2C Fig). Additionally, the biliary duct network in *lvh* labeled by the *Tg(tp1*:*Tomato)*^*cq109*^ transgene and blood vessel labeled by the *Tg(kdrl*:*mCherry)*^*cq15*^ transgene were relatively normal (S2D–S2G Fig). These results show that the fasting-induced hypertrophy in *lvh* is specific to the liver.

### The *lvh* mutant exhibits liver dysfunction under fasting and limited tolerance to starvation

**Fig 2:**
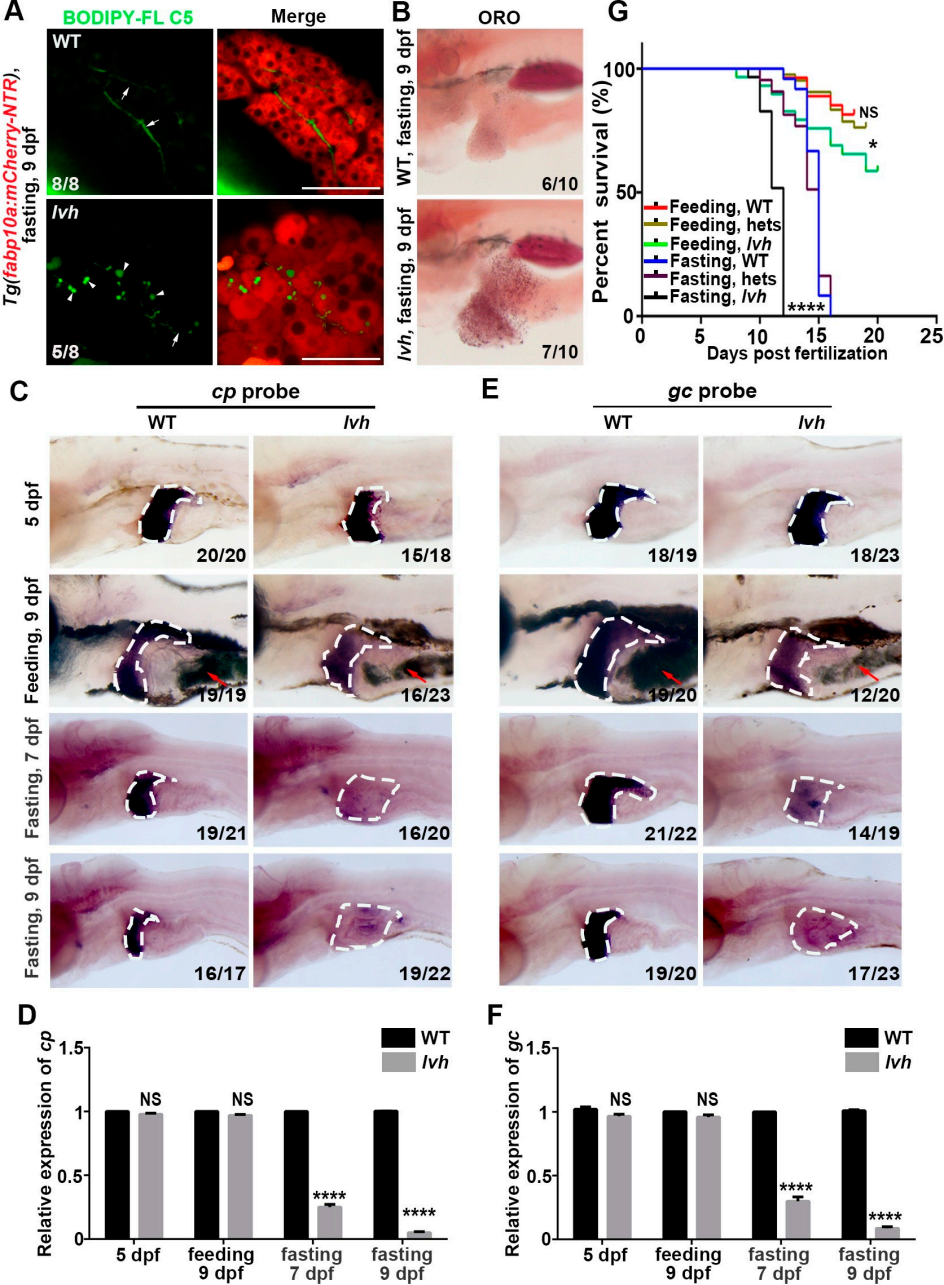
The *lvh* mutant exhibits liver dysfunction under fasting and limited tolerance to starvation. (A) Double labeling of mCherry and BODIPY FL C5 epifluorescence in the wild-type (8/8) and *lvh* (5/8) liver at 9 dpf. Arrows and arrowheads indicate intrahepatic ducts and lipid droplets, respectively. (B) ORO staining showing fat deposits in the liver of wild-type and *lvh* at 9 dpf. (C) Expressions of *cp* in the wild-type and *lvh*. The dashed frames indicate the liver area. The red arrowheads indicate food in the gut. (D) qPCR data showing the relative expression levels of *cp*. (E) Expressions of *gc* in the wild-type and *lvh*. The dashed frames indicate the liver area. The red arrowheads indicate food in the gut. (F) qPCR data showing the relative expression levels of *gc*. (G) The survival rates of wild-type, hets and, *lvh* under feeding or fasting are shown by the Gehan-Breslow-Wilcoxon test. Asterisks indicate statistical significance. NS, not significant. *P<0.05, ****P<0.0001. Data are represented as mean±SD. WT, wild-type. hets, heterozygosity. Scale bars, 50 μm.

To analyze whether the *lvh* hepatocytes are functional, we performed fluorescent BODIPY FL C5 assay, which is a fatty acid analogue. This assay allows visualization of fatty acid metabolism as well as hepatocyte bile secretion and the bile-conducting function of bile ducts [29]. The *lvh* mutant exhibited defective bile secretion and accumulations of lipid droplets (Fig 2A). The oil red O (ORO) lipid staining assay indicated a substantial amount of lipid accumulation in *lvh* liver (Fig 2B). We checked the expressions of critical genes involved in lipid metabolism and transport and found that the fatty acid oxidation relation genes (*cpt1aa*, *cpt1ab*, and *acsl2*) were up-regulation in *lvh* liver, and lipolysis relation gene *lipca* was up-regulation. However, we found some genes related to lipid transport (*cetp* and *mttp*) were significantly downregulated in the *lvh* liver (S3A–S3C Fig). The *angiopoietin-like 3 (angptl3)*, which has been reported for zebrafish liver lipid metabolism [30], was also significantly downregulated in the *lvh* liver (S3B–S3C Fig). Those data indicate that lipid metabolism and transport are abnormal in *lvh* liver.

In contrast to siblings, the expressions of hepatocyte functional markers vitamin D binding protein (*gc*) and ceruloplasmin (*cp*) [31] in *lvh* maintained under feeding, but nearly disappeared under fasting (Fig 2C–2F), suggesting that the hepatomegaly of *lvh* is accompanied by liver dysfunction. When food deprivation was prolonged, most of the *lvh* mutants died at 11–12 dpf, whereas most of the siblings died at 13–14 dpf and could even survive to 15 dpf (Fig 2G). About half of the *lvh* mutants survived to adulthood under feeding (Fig 2G). All these results demonstrate liver dysfunction under fasting and defective response to energy stress in the *lvh* mutant.

### The hepatomegaly phenotype of *lvh* is caused by mutation of *ftcd*

**Fig 3:**
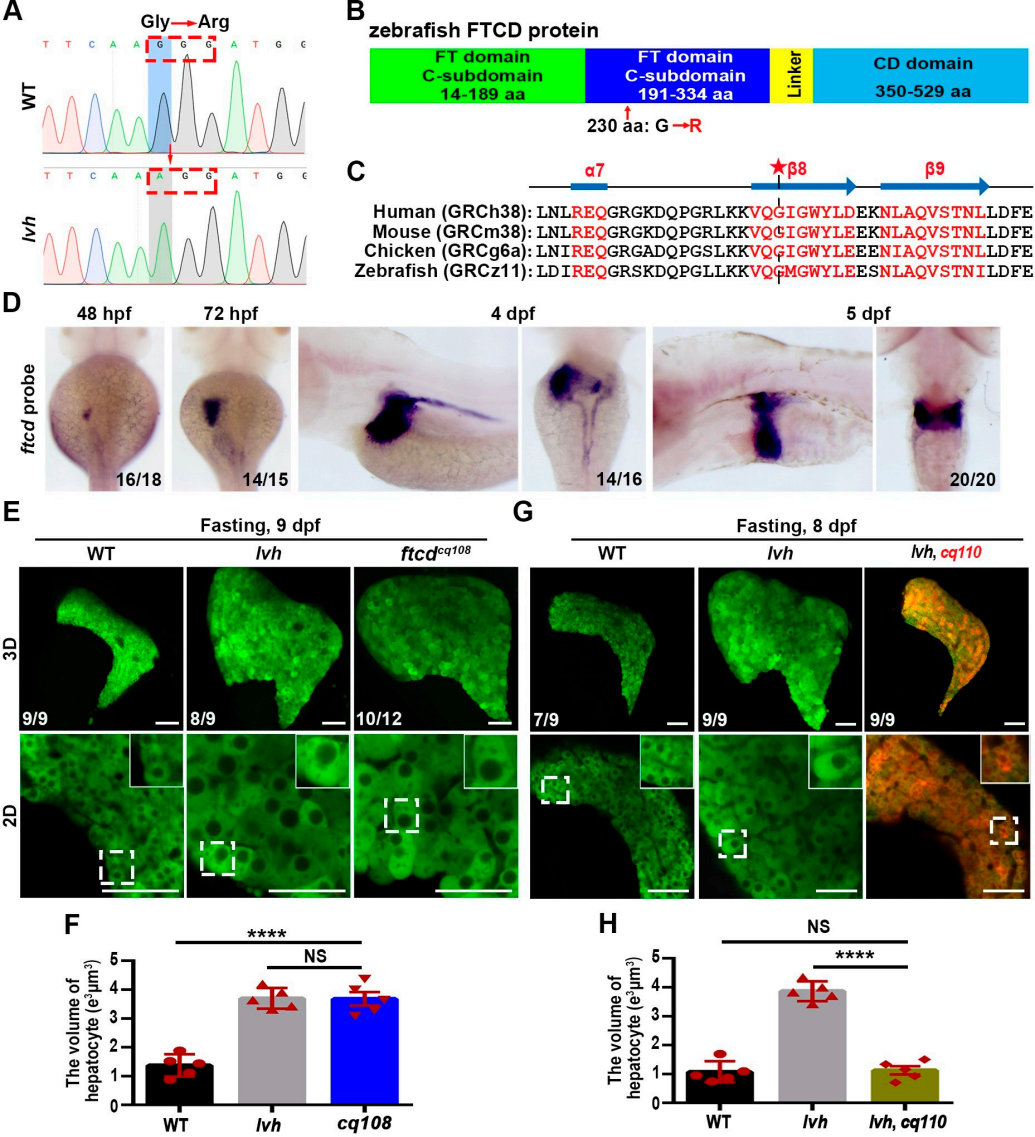
The hepatomegaly of *lvh* is caused by mutation of *ftcd*. (A) Sequencing of *ftcd* from the wild-type and *lvh* mutant identified a G to A point mutation, resulting in a switch from glycine to arginine at position 691. Red arrow indicates the mutation site. (B) Diagramed FTCD protein structure and the mutation in *lvh* at FT domain C-subdomain. (C) A conserved β8 motif of FTCD in zebrafish and its homologous proteins in humans, mouse and chicken. Asterisk indicates the site of mutated amino acid in *lvh*. (D) WISH images showing *ftcd* expressions at 48 hpf, 72 hpf, 4 dpf, and 5 dpf. (E) 3D confocal projection and 2D single-optical section images showing the liver of wild-type, *lvh* and, *ftcd*^*cq108*^ at 9 dpf. Higher magnification images of single hepatocytes are displayed. (F) Unpaired Student’s *t*-test for single hepatocyte volume in the wild-type (n = 5), *lvh* (n = 5) and *cq*^*108*^ (n = 5). (G) 3D confocal projection and 2D single-optical section images showing the liver of WT, *lvh* and *lvh* under *cq*^*110*^ background at 8 dpf. Higher magnification images of single hepatocytes are displayed. (H) Unpaired Student’s *t-*test for single hepatocyte volume in the wild-type (n = 5), *lvh* (n = 5) and *lvh* under *cq*^*110*^ (n = 5). Asterisks indicate statistical significance. ****P<0.0001. Data are represented as mean±SD. WT, wild-type. Scale bars, 50 μm.

Positional cloning indicated that *lvh* contained a G to A mutation in the sixth exon of *ftcd* gene on chromosome 22 (Figs 3A and S4A), which led to an amino acid switch from glycine to arginine (Fig 3A). The mutated glycine was located in the FT domain’s C-subdomain and at the eighth β-motif of FTCD, which was highly conserved among vertebrates [32] (Fig 3B and 3C). The expression of *ftcd* was enriched in the liver from 48 hours post fertilization (hpf), and accumulated in the liver and pronephric duct from 4 dpf, then mainly localized in the liver since 5 dpf (Fig 3D). The *ftcd* remained expressed in the liver under fasting (S4B Fig).

We designed two Cas9 targets (S5A Fig) and verified both were working (S5B Fig). An independent *ftcd*^*cq108*^ mutant allele was generated by CRISPR/Cas9 and single *ftcd* gRNA, which carried a 6-base pair deletion in the sixth exon (S5C Fig) and alteration of the eighth β-motif of FTCD (S5C Fig). Using CRISPR/Cas9 and two different *ftcd* gRNA, we gained a *ftcd*^*L-cas9*^ large fragment knockout mutant (S5D Fig). *ftcd*^*cq108*^ and *ftcd*^*L-cas9*^ photocopied the hepatomegaly and enlarged hepatocyte size phenotypes of *lvh* under fasting (Figs 3E, 3F and S5E). Furthermore, the full-length *ftcd* CDS was amplified through 5’-RACE (rapid-amplification of cDNA ends) because of the incomplete zebrafish *ftcd* 5’-CDS in the GRCz11 database (http://ensembl.org/Danio_rerio/Gene/Summary?db=core;g=ENSDARG00000007421;r=22:12770877-12786532;t=ENSDART00000044683) (S6A Fig). Then, the *Tg(fabp10a*: *ftcd-p2A-DsRed)*^*cq110*^ transgenic line was generated to specifically overexpress FTCD in hepatocytes (S6B Fig), which rescued the fasting-induced hepatomegaly of *lvh* (Figs 3G, 3H and S6B) and did not affect liver function (S6C Fig). These results validate that the *lvh* phenotypes are caused by the mutation of *ftcd* and indicate protection of liver from the fasting-induced hepatomegaly by FTCD.

### FTCD downregulates mTORC1 signaling in response to starvation

**Fig 4:**
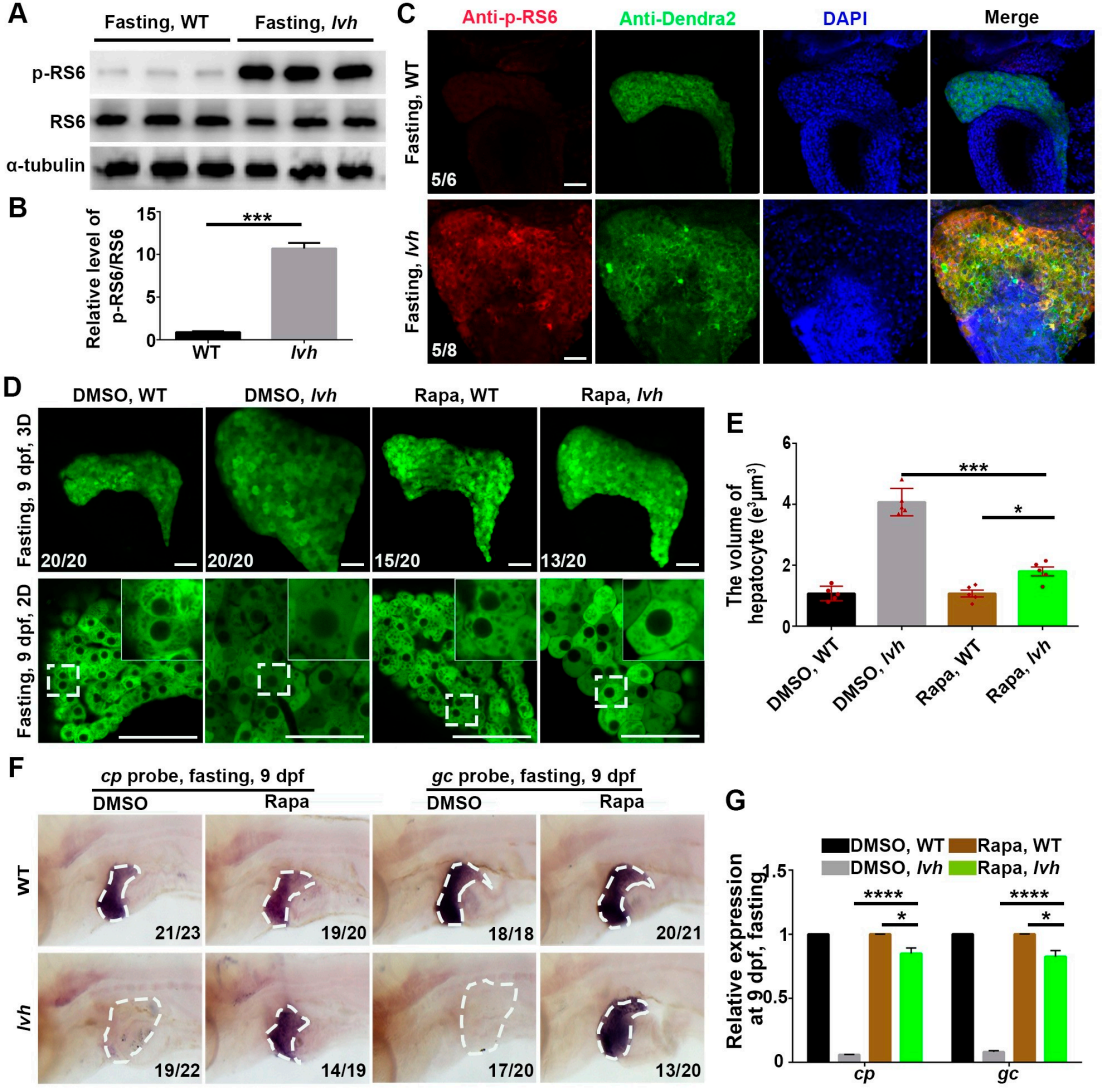
FTCD prevents starvation-induced hepatomegaly through downregulating mTORC1. (A) Western blot analysis of p-RS6 (s240/244), RS6, and α-tubulin using liver lysates (n = 120). (B) Quantification of relative intensity (n = 3) for p-RS6/RS6. (C) Immunostaining for p-RS6 and Dendra2 in livers (3D imaging). Nuclei were stained with DAPI (blue). (D) 3D confocal projection and 2D single-optical section images of the liver. Higher magnification images of single hepatocytes are displayed. (E) Unpaired Student’s *t*-test for single hepatocyte volume of DMSO and Rapa treatment in the wild-type (n = 5) and *lvh* (n = 5). (F) The disappeared *cp* and *gc* expressions in *lvh* under fasting were rescued by rapamycin treatment. The dashed boxes indicate the liver area. (G) qPCR data showing the relative expression levels of *cp* and *gc* in the wild-type and *lvh* liver after DMSO and Rapa treatment. Asterisks indicate statistical significance. NS, not significant. *P<0.05, ***P<0.001, ****P<0.0001. Data are represented as mean±SD. WT, wild-type. Rapa, rapamycin. Scale bars, 50 μm.

The mTORC1 signaling plays vital roles in regulating liver energy balance [8], and its downregulation is implicated in reducing cell size under starvation [10]. To investigate the relationship between FTCD and mTORC1 signaling, the livers were dissected and the levels of phosphorylated ribosomal S6 protein (p-RS6), a well-known downstream effector of mTORC1, were analyzed. In contrast to the WT, the level of p-RS6 remained at high levels in the liver of *lvh* under fasting (Fig 4A–4C). These results suggest FTCD as an important factor to downregulate mTORC1 in response to starvation.

We then investigate whether inhibition of mTORC1 signaling could rescue *lvh* phenotypes. Treatment of rapamycin, a specific mTORC1 inhibitor [33], from 5 dpf to 9 dpf rescued the fasting-induced hepatomegaly and enlarged hepatocyte size of *lvh* at 9 dpf (Fig 4D and 4E). Furthermore, rapamycin treatment restored the expressions of functional hepatocyte markers *cp* and *gc* in *lvh* liver under fasting (Fig 4F and 4G), suggesting rescues of liver functions. These results demonstrate that in response to starvation, FTCD acts as an important upstream factor to downregulate the mTORC1 signaling, thus protecting the liver from starvation-induced liver hypertrophy and dysfunction.

## Discussion

In response to starvation stress, the liver initiates a series of metabolic adaptations to maintain energy homeostasis, and mTORC1 pathway is downregulated to ensure adequate usage of limited resources [3,8]. In this study, we identified an *lvh/ftcd* mutant with relatively normal liver under feeding but exhibiting hepatomegaly under fasting, thus revealing previously unexpected roles of FTCD in the starvation-induced response through downregulating mTORC1. FTCD can catalyze two reactions in the histidine degradation pathway. Using L-histidine, D-histidine, and folic acid to interfere with the metabolism of histidine [34], we did not observe hepatomegaly in WT (S7 Fig). Meanwhile, we assessed the expression of LC3B and found the LC3B-I and LC3B-II were both down-regulated in *lvh* liver (S8 Fig). The level of autophagy is upregulated for the maintenance of energy homeostasis in the liver during starvation [35], and activation of mTORC1 signaling inhibits autophagy [36]. Recent literature reveals that FTCD has a structural role in tethering biological membranes other than enzymatic function [37]. Thus, FTCD might promote the formation of autophagosomes due to its structural functions, then feedback to downregulate the mTORC1 pathway or might affect the localization of mTORC1 complex to regulate mTORC1 activity in cells. However, these speculations require further exploration and verification.

In HEK293T cells and HepG2 cells, the change of p-RS6 level in starvation was lower after *FTCD* knockdown than control group (S9 Fig), indicating FTCD is also a key factor in regulating mTORC1 to response energy in mammalian cells. The ratio of LC3B-II / LC3B-I was lower in FTCD-knockdown HEK293T cells compared to control cells, indicating some difference from the zebrafish to mammalian cells, which may be caused by the microenvironment difference between *in vitro* and *in vivo*. The phosphoinositide 3-kinase (PI3K)/protein kinase (AKT) signaling pathway and the adenosine monophosphate-activated protein kinase (AMPK) signaling pathway are the positive and negative upstream regulatory for mTORC1, respectively [38,39]. In HEK293T cells with *FTCD* knockdown, the relative levels of p-AKT/AKT and p-AMPK/AMPK were slightly changed under starvation compared with the control siRNA group (S9B and S9C Fig). The detailed molecular mechanisms underlying the effect of FTCD on AKT and AMPK phosphorylation need to be further investigated.

Traditionally, the FTCD deficiency causes glutamate formiminotransferase deficiency (FIGLU-uria or FTCD deficiency), which is the second most common inborn defect of folate metabolism [23,24]. The patient may exhibit severe mental retardation and megaloblastic anemia in the early-reported cases [40,41]. Later, some FTCD deficiency patients with possible mild developmental delay and no hematologic abnormalities were reported [42,43]. Among all the FTCD deficiency patients, the cases displaying symptoms are limited. Recently, a tandem mass spectrometry-based newborn screen suggested that the majority of individuals with FTCD deficiency were asymptomatic [44]. That is consistent with the *ftcd* mutant zebrafish under normal feedings. Nevertheless, our findings suggest that FTCD deficiency patients may suffer from extra hazards during starvation.

FTCD serves as an immunohistochemical tumor marker and is a candidate tumor suppressor in hepatocellular carcinoma (HCC) [21,22]. However, the functions of FTCD in HCC remain unknown. FTCD is down-regulated in HCC [22], whereas the mTORC1 pathway is frequently upregulated there [45]. Our findings reveal downregulation of mTORC1 by FTCD, which provides potential explanations on the roles of FTCD in HCC. Taken together, this study revealed the roles of FTCD in the starvation-induced energy metabolism in the liver through downregulating mTORC1, providing further potential clinical warn to FTCD deficiency patients as well as a potential target for the HCC clinical studies.

## Materials and methods

### Ethics statement

All experimental procedures were performed according to the standard guidelines and approved by Southwest University (Chongqing, China). Zebrafish was maintained according to the Guidelines of Experimental Animal Welfare from the Ministry of Science and Technology of China (2006) and the Institutional Animal Care and Use Committee protocols from Southwest University (2007).

### Zebrafish strains

The *Tg(fabp10a*:*Dendra2-NTR)*^*cq1*^, *Tg(fabp10a*:*mCherry-NTR)*^*cq2*^, *Tg(kdrl*:*mCherry)*^*cq15*^, *Tg(Ras*:*GFP)* [46], and *Tg(-3*.*5ubb*:*Cerulean-gmnn-2A-mCherry-cdt1)* abbreviated as Fucci [47], transgenic lines were used. We used *lvh*^*cq107*^, *ftcd*^*cq108*^, *ftcd*^*L-cas9*^, mutant and used the polymorphic line SJD as a genetic mapping strain. The methods of generation of *Tg(tp1*:*Tomato)* and *Tg(fabp10a*:*ftcd-p2a-DsRed)* transgenic lines used previously described [27]. Embryos were treated with 0.003% 1-phenyl-2-thiourea (PTU, Sigma-Aldrich, Darmstadt, Germany) from 24 hours post-fertilization to inhibit pigmentation for In situ hybridizations, antibody stainings, and imaging experiments. All zebrafish lines were raised and maintained under standard laboratory conditions according to institutional animal care and used committee protocols as previously described [48,49].

### ENU mutagenesis

N-ethyl-N-nitrosourea (ENU) (Sigma, USA) mutagenesis was carried out as previously described [50]. The twelve adult male zebrafish of *Tg(fabp10a*:*Dendra2-NTR)* were treated with 3.5 mM ENU for 1 h at weekly intervals and repeated for six times. Two weeks after the ENU treatment, those male zebrafish were outcrossed to wild-type females to generate F1 families. F1 outcross with *Tg(fabp10a*:*Dendra2-NTR)* line after sexual maturity to generate F2 families. F2 siblings were intercrossed to generate F3 embryos for screening. The 180 F2 families were produced, and now we have screened 72. We analyzed the liver morphology under fasting in F3 embryos to identify the mutants, and have identified seven positives mutants.

### Whole-mount *in situ* hybridization, antibody stainings, and imaging

Whole-mount in situ hybridization (WISH) was performed as previously described [31]. The primers used to amplify for probe synthesis are listed in Table A in S1 Table. Then PCR products were used to generate probes using the DIG RNA labeling Kit (Roche Applied Science, Penzberg, Germany). The WISH images were captured using the SteREO Discovery V20 microscope (Carl Zeiss, Germany).

Whole-mount antibody staining was performed as previously described [51]. Primary antibodies anti-pRS6 (S240/244) (1:500; #2215 Cell Signaling, USA), anti-LC3B (1:1000; ab192890, Abcam), anti-Dendra2 (1:1000, AB821, Evrogen, Moscow, Russia). Secondary antibodies used in the study are donkey anti-goat IgG Alexa fluor 568-conjugated (1:1000, Invitrogen) and donkey anti-rabbit IgG Alexa fluor 488-conjugated (1:1000, Invitrogen). Images were captured using ZEN2010 software equipped on an LSM880 confocal microscope (Carl Zeiss).

### BODIPY assay and staining

Larvae were fed with BODIPY FL C5 (Invitrogen, Grand Island, NY) for 4 hours as previously described [29]. Then larvae were washed with egg water 3 times and subsequently live imaging.

### Oil Red O staining

Add 0.5 g Oil Red O (ORO) (BBI Life Sciences) to 100 ml 100% isopropyl alcohol as ORO stock solution. Larvae were fixed with 4% PFA at 4°C overnight. The larvae were washed with PBS for 3 times, then washed with 60% isopropanol for 1 hour, and subsequently stained with filtered ORO working solution (3 part ORO stock solution to 2 part H_2_O) for 15 min at room temperature, and then washed twice with H_2_O for 15 min.

### Positional cloning of *lvh* mutation gene

Heterozygous *lvh* fish were outcrossed with the SJD line to generate the mapping population. Subsequent mapping was performed as previously described [28,52]. Using SSLP (simple sequence length polymorphism) Luo lab Z markers in bulked segregant analysis, we found that *lvh* mutation linked to chromosome 22. Recombinant analysis of 538 mutant embryos further narrowed the location of the *lvh* mutation to a 0.37 Mbp (million base pairs) genomic DNA fragment, which contains 11 genes (*adarb1a*, *fam207a*, *plcd4a*, *cnot9*, *ftcd*, *mstna*, *stat1a*, *glsa*, *nab1a*, *mfsd6a*, and *ahr1b*). The mutant genotype was finally identified by sequencing the PCR fragment containing the coding sequences of the 11 genes.

### CRISPR/Cas9 system-mediated mutagenesis

The CRISPR/Cas9 system was performed as previously described [53]. The generation of *ftcd* cas9 mutant was depicted in S5A Fig. In brief, CRISPR/Cas9 target sites was designed in the 6th exon and the 1st exon. The gRNA (200–300 ng/μl) and Cas9 mRNA (300 ng/μl) were co-injected into 1-cell stage *Tg(fabp10a*:*Dendra2-NTR)* embryos, and then lysate of 15–30 embryos at 48–72 hpf was used as templates for PCR. The primers used for checking sequences or synthesizing gRNA are listed in Table B in S1 Table. Embryos with effective genome editing were raised to adults (F0), and then F0 outcrossed with *Tg(fabp10a*:*Dendra2-NTR)* to generate F1. The individual of F1 adults used the tail fin genomic DNA as templates to do PCR and sequence, then the genotypes of mutants were determined by DNA sequencing.

### Western blot

The zebrafish livers at fasting 9 dpf and cultured HEK293T cells were lysed using RIPA lysis. For Western blot as previously described [54,55]. The protein samples was resolved on a 10% or 12% SDS-PAGE gel, then transferred onto a PVDF membrane (Millipore). Western blot was performed using anti-pRS6 (S240/244) (1:1000; #2215 Cell Signaling, USA), anti-RS6 (1:1000; #2217 Cell Signaling, USA), anti-LC3B (1:1000; ab192890, Abcam), anti-pAKT(Ser473) (1:500; #4046 Cell Signaling, USA), anti-AKT (1:1000; #4691 Cell Signaling, USA), anti-FTCD (1:200; #93327 Cell Signaling, USA), anti-pAMPKα(Thr172) (1:500; #2535 Cell Signaling, USA), anti-AMPKα (1:1000; #2532 Cell Signaling, USA), anti-α-tubulin (1:2000, Santa Cruz, USA), anti-mouse-HRP (1:2000, Abcam, UK) and anti-rabbit-HRP (1:2000, Abcam, UK).

### Rapamycin treatment

For rapamycin treatment, larvae were treated with 10 μM rapamycin (Selleck) in 0.2% DMSO. Every 24 hours, the larvae were added newly prepared rapamycin solution or washed three times and recovered in egg water.

### Assessment of survival rate

Using the heterozygosity of *lvh* mutant to cross, and their embryos were randomly assigned to four dishes with 50 embryos each. At 5 dpf, two dishes larvae transferred to two incubators and be fed every 12 hours. The other two dishes larvae were fasted. From 5 dpf to 24 dpf, every 12 hours, observed those larvae, counted the dead larvae, extracted individual genomes, did PCR to sequence, and analyzed their genotypes. At 24 dpf, the genomes of each remaining individual were extracted and did PCR to sequence. Finally, the survival rates of different genotypes were determined.

### Folic acid, D-Histidine, and L-Histidine treatment

The Folic acid, D-Histidine, and L-Histidine treatment purchased from Sangon Biotech (Shanghai, China), were configured in different concentrations by egg-water. Larvae were treated from 5 dpf to 9 dpf, and changed the treatment solution every 24 hours.

### Quantitative real-time polymerase chain reaction (qPCR)

Total RNA was extracted from 100 dissected livers using Tripure isolation reagent (Roche, Indianapolis, IN). All liver tissues were manually dissected from the WT larvae or *lvh* at different times. cDNA was synthesized using Omniscript RT Kit (Qiagen, Valencia, CA) and qPCR was performed using the FastStart Universal SYBR Green Master (Roche). The primers used for qPCR are listed in Table C in S1 Table.

### Cell culture and transfection

HEK293T cell line was purchased from the Cell Bank, Chinese Academy of Sciences (Shanghai, China), and HepG2 cell line (a gift from Prof. Zhan Lei, Southwest University), both cultured in Dulbecco’s modified Eagle’s medium (DMEM) supplemented with 10% fetal bovine serum (FBS). All the cells were maintained at 37°C in 5% CO_2_-containing atmosphere. Cell starvation was treated with Earle’s Balanced Salts Solution (EBSS) 4 hours. The medium and serum were purchased from Gibco.

Human FTCD siRNA: 5’-GCUGGUACCUGGAUGAGAATT-3’ and synthesized by Genomeditech (Shanghai, China), The siRNA (50 nM) was transfected into cells using Lipofectamine 3000 transfection reagent (Invitrogen).

### Quantification and statistical analysis

All experiments and samples selection are performed blinded. Antibody stained and imaged using ZEN 2012 software equipped on an LSM880 confocal microscope (Carl Zeiss). We used the Imaris 6.0 software to do 3D reconstruction and statistical analysis of nucleus and volume. All figures, labels, arrows, scale bars, and outlines were drawn using the Adobe Photoshop software. The exact sample number (n), *P* value for each experimental group and statistical tests were indicated in the figure legends. All numeric data are presented as mean ± SD. Differences between the two groups were evaluated using the Unpaired Student’s t test. All statistical analyses were performed using GraphPad Prism 6.0 software, and *P* < 0.05 was considered statistically significant.

## Supporting information

S1 Fig The *lvh* mutant exhibits hepatomegaly under fasting. (A) Bright-field and fluorescent images showing the hepatomegaly of *lvh* under fasting, with the body shape unaffected. (B) 3D confocal projection images showing the progress of hepatomegaly development in *lvh* under fasting from 5 dpf to 11 dpf. WT, wild-type. Scale bars, 50 μm.
(TIF)

S2 Fig The gut and pancreas are unaffected in *lvh* under fasting. (A-C) WISH of *ifabp* (A), *insulin* (B), and *trypsin* (C) showing that the gut, pancreatic *β* cell, and exocrine pancreas are unaffected in *lvh* under fasting. (D) Confocal images indicating the liver and bile duct network of wild-type and *lvh* at 9 dpf. White brackets indicate the diameters of the bile duct. (E) Unpaired Student’s *t*-test for the diameters of the bile duct in wild-type (n = 10) and *lvh* (n = 10). (F) Confocal images indicating the liver and blood vessel network of wild-type and *lvh* at 8 dpf. White brackets indicate the diameters of the blood vessel. (G) Unpaired Student’s *t*-test for the diameters of the blood vessel in wild-type (n = 10) and *lvh* (n = 10). NS, not significant. Data are represented as mean±SD. WT, wild-type. Scale bars, 50 μm.
(TIF)

S3 Fig Some genes related to lipid metabolism exhibits abnormal in the *lvh* mutant. (A) qPCR data showing the relative expression levels of fatty acid oxidation relation genes *(cpt1aa*, *cpt1ab*, *acs1b*, *acsl2)* and lipolysis relation genes (*mgll*, *lpl*, *lipca*, *lipcb*) in the WT and *lvh* liver. (B) qPCR data showing the relative expression levels of lipid transport relation genes (*apoa2*, *cettp*, *mttp*) and *angptl3* that acts upstream of or within lipid metabolic process in zebrafish. (C) WISH images showing the expressions of *apoa2*, *cettp*, *mttp*, and *angptl3* in WT and *lvh* at fasting 9 dpf. WT, wild-type.
(TIF)

S4 Fig Position cloning and expression of the mutated gene of *lvh*. (A) Position cloning narrows down the mutated gene of *lvh* to chromosome 22 (blue line) in a region including the *ftcd* locus. (B) WISH images showing the expression pattern of *ftcd* at 7 dpf and 9 dpf under both feeding and fasting. The red arrowheads indicate food in the gut.
(TIF)

S5 Fig Generation of *ftcd* mutants using CRISPR/Cas9. (A) The schematic illustrations of *ftcd* mutant generation using the CRISPR/Cas9 system. The sgRNA target and PAM region are displayed in green and red, respectively. (B) The two Cas9 target sites for knockout *ftcd* gene worked in knock out F0 generation. (C) A *ftcd* Cas9 mutant line by the single-target site generation and the FTCD protein sequences of wild-type, *lvh*, and *ftcd*^*cq108*^ at the β8 motif region. (D) A large fragment knockout Cas9 mutant of *ftcd* by two target sites generation. (E) Bright-field and fluorescent images showing the hepatomegaly phenotype of *ftcd*^*cq108*^ mutant and *ftcd*^*L-ca9*^, with the body shape unaffected. WT, wild-type.
(TIF)

S6 Fig Overexpression of *ftcd* in the liver rescues the *lvh* mutant phenotype. (A) Obtain of ftcd full-length CDS sequence by 5’-RACE. (B) Bright-field and fluorescent images of *lvh* with or without *ftcd* overexpression.(C) WISH images showing the expressions of *cp*, *gc*, *cept*, *and angptl3* in WT with or without *ftcd* overexpression. WT, wild-type.
(TIF)

S7 Fig Using chemicals to interfere with the FTCD enzymatic reaction. (A) Experimental scheme illustrating the stage of chemicals treatment to wt and *lvh*. (B) Bright-field and fluorescent images showing the liver morphology of wt and *lvh* were treated with folic acid, D-His or L-His. (C) 3D confocal projection images showing the liver morphology of wt and *lvh* were treated with folic acid, D-His, or L-His at fasting 9 dpf. WT, wild-type. Scale bars, 50 μm.
(TIF)

S8 Fig Autophagy levels were reduced in the liver of *lvh*. (A) Western blot analysis of LC3B using wt and *lvh* liver lysates (n = 120). (B) Immunostaining for LC3B and Dendra2 in livers of wt and *lvh* at fasting 9 dpf (2D imaging). Nuclei were stained with DAPI (blue). (C) Unpaired Student’s *t*-test for the number of LC3B positive spots in the wild-type (n = 5) and *lvh* (n = 5). WT, wild-type. Scale bars, 50 μm.
(TIF)

S9 Fig The function of FTCD is partially conserved in HEK293T and HepG2 cells. (A) In HEK293T cells, knockdown of *FTCD* by siRNA duplexes. After transfection for 48 h, the cells were analyzed by Western blotting with antibodies to FTCD and a-tubulin. (B) Western blot analysis of p-RS6, RS6, P-AKT, AKT, P-AMPK, AMPK, and LC3B use HEK293T cells transfected with control siRNA or *FTCD* siRNA and without starvation (Control) or starved for 4 h. (C) The relative band intensity of the Western blot in B. (D) In HepG2 cells, knockdown of *FTCD* by siRNA duplexes. After transfection with 48 h, the cells were analyzed for Western blotting with antibodies to FTCD and a-tubulin. (E) Western blot analysis the expressions of p-RS6 and RS6 using HepG2 cells transfected with control siRNA or *FTCD* siRNA under starvation (4 h) and control. (F) Relative band intensity of p-RS6/RS6 in E.
(TIF)

S1 Table Primer sequences were used for this study. (DOCX)

S1 File All numerical data in this study. (XLSX)
