# Supplementary figures and images for: Formimidoyltransferase cyclodeaminase prevents the starvation-induced liver hepatomegaly and dysfunction through downregulating mTORC1
Source: PLoS Genet. 2021 Dec 23;17(12):e1009980. doi: 10.1371/journal.pgen.1009980 (PMC8741050; doi:10.1371/journal.pgen.1009980)

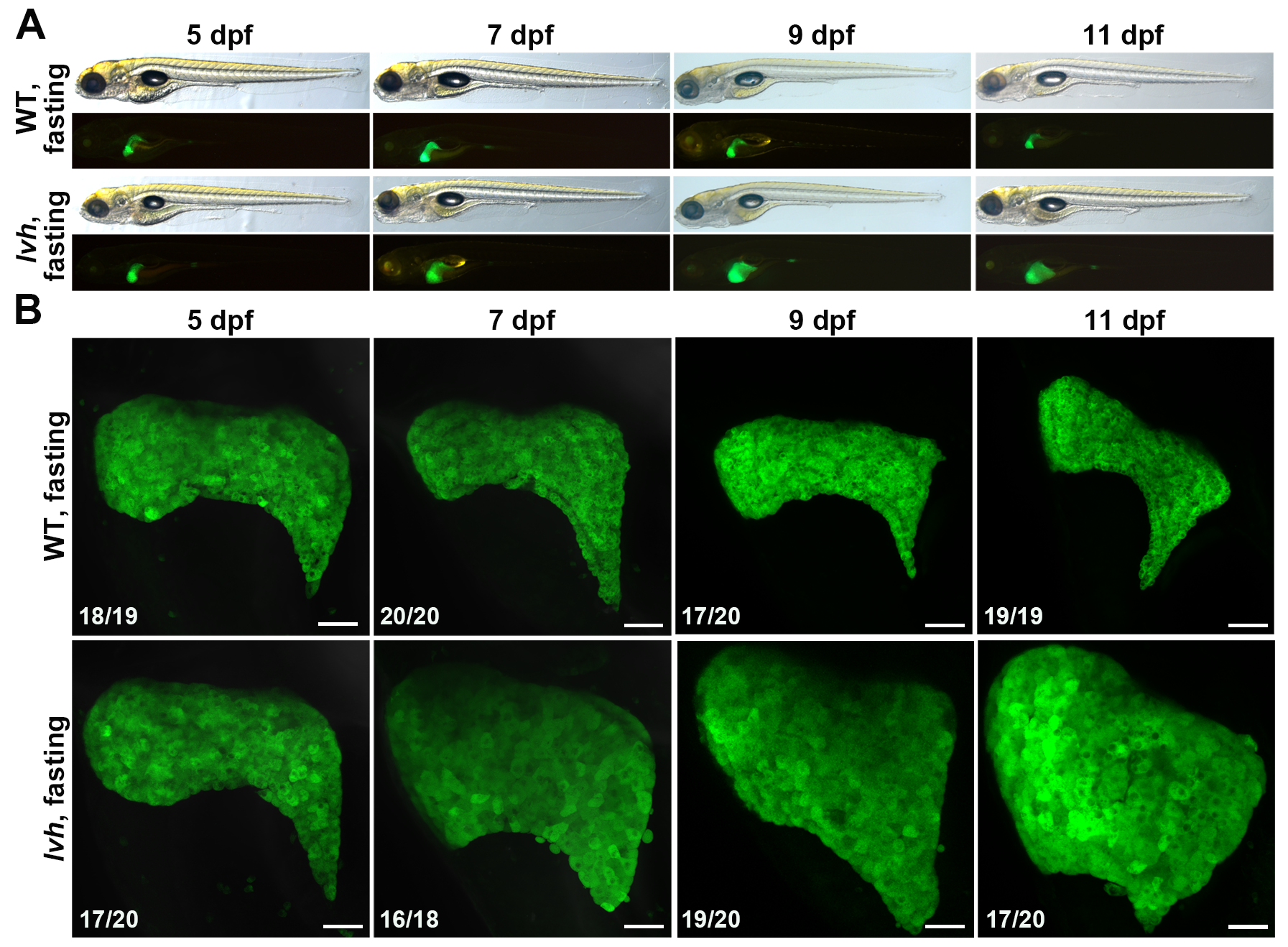

Supplement: S1 Fig — (A) Bright-field and fluorescent images showing the hepatomegaly of lvh under fasting, with the body shape unaffected. (B) 3D confocal projection images showing the progress of hepatomegaly development in lvh under fasting from 5 dpf to 11 dpf. WT, wild-type. Scale bars, 50 μm. (TIF) [file pgen.1009980.s001.tif]

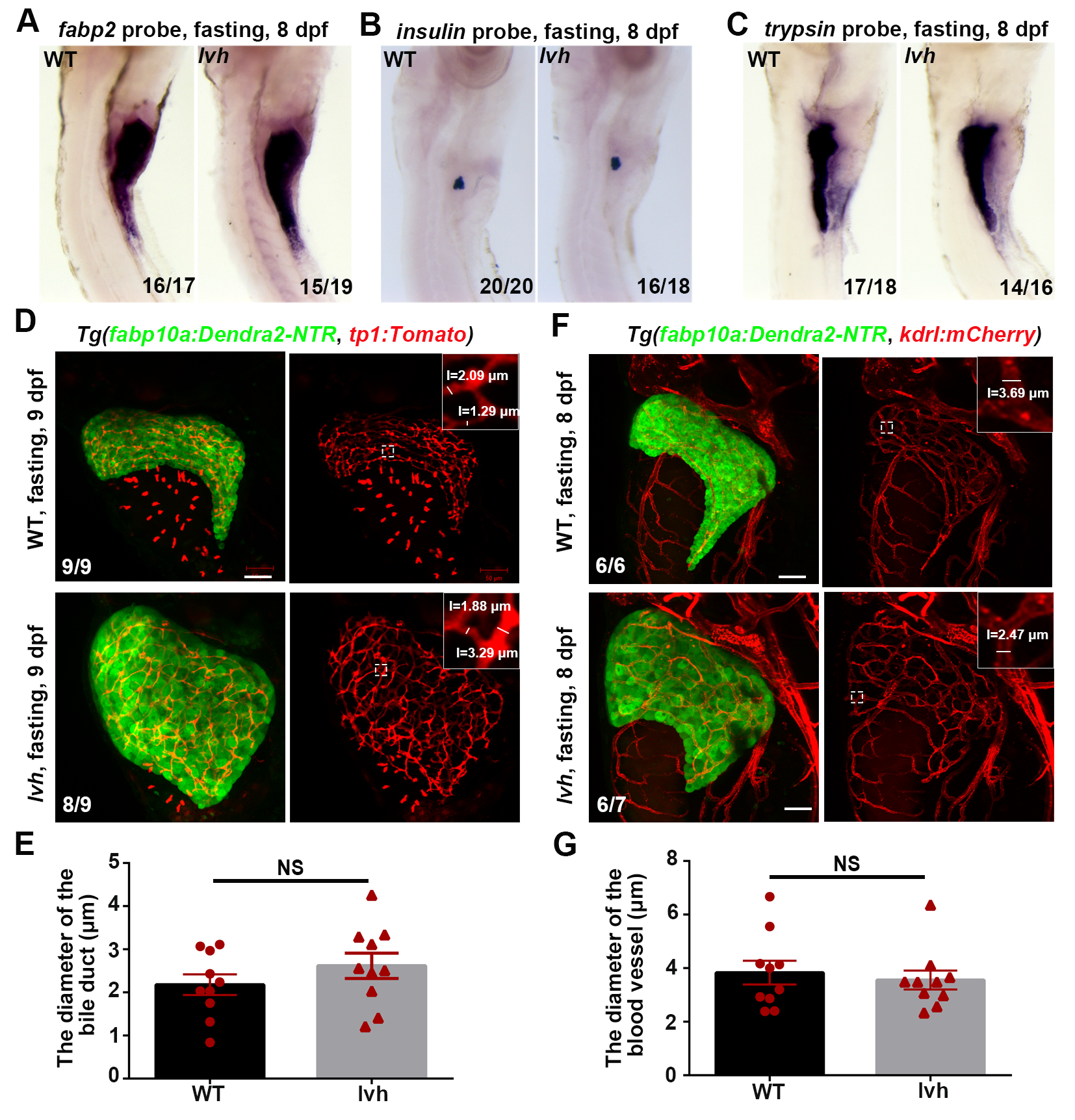

Supplement: S2 Fig — (A-C) WISH of ifabp (A), insulin (B), and trypsin (C) showing that the gut, pancreatic β cell, and exocrine pancreas are unaffected in lvh under fasting. (D) Confocal images indicating the liver and bile duct network of wild-type and lvh at 9 dpf. White brackets indicate the diameters of the bile duct. (E) Unpaired Student’s t-test for the diameters of the bile duct in wild-type (n = 10) and lvh (n = 10). (F) Confocal images indicating the liver and blood vessel network of wild-type and lvh at 8 dpf. White brackets indicate the diameters of the blood vessel. (G) Unpaired Student’s t-test for the diameters of the blood vessel in wild-type (n = 10) and lvh (n = 10). NS, not significant. Data are represented as mean±SD. WT, wild-type. Scale bars, 50 μm. (TIF) [file pgen.1009980.s002.tif]

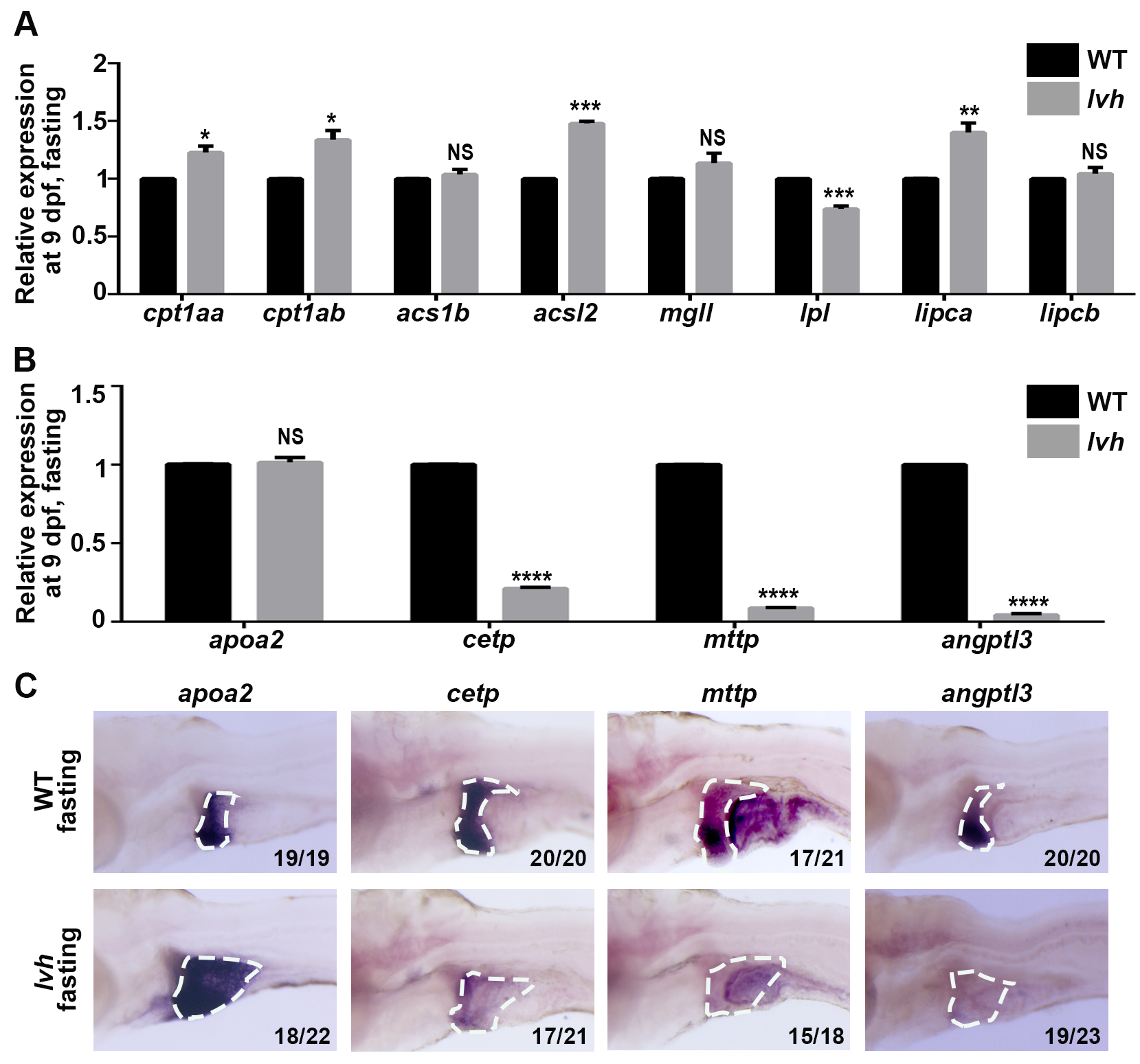

Supplement: S3 Fig — (A) qPCR data showing the relative expression levels of fatty acid oxidation relation genes (cpt1aa, cpt1ab, acs1b, acsl2) and lipolysis relation genes (mgll, lpl, lipca, lipcb) in the WT and lvh liver. (B) qPCR data showing the relative expression levels of lipid transport relation genes (apoa2, cettp, mttp) and angptl3 that acts upstream of or within lipid metabolic process in zebrafish. (C) WISH images showing the expressions of apoa2, cettp, mttp, and angptl3 in WT and lvh at fasting 9 dpf. WT, wild-type. (TIF) [file pgen.1009980.s003.tif]

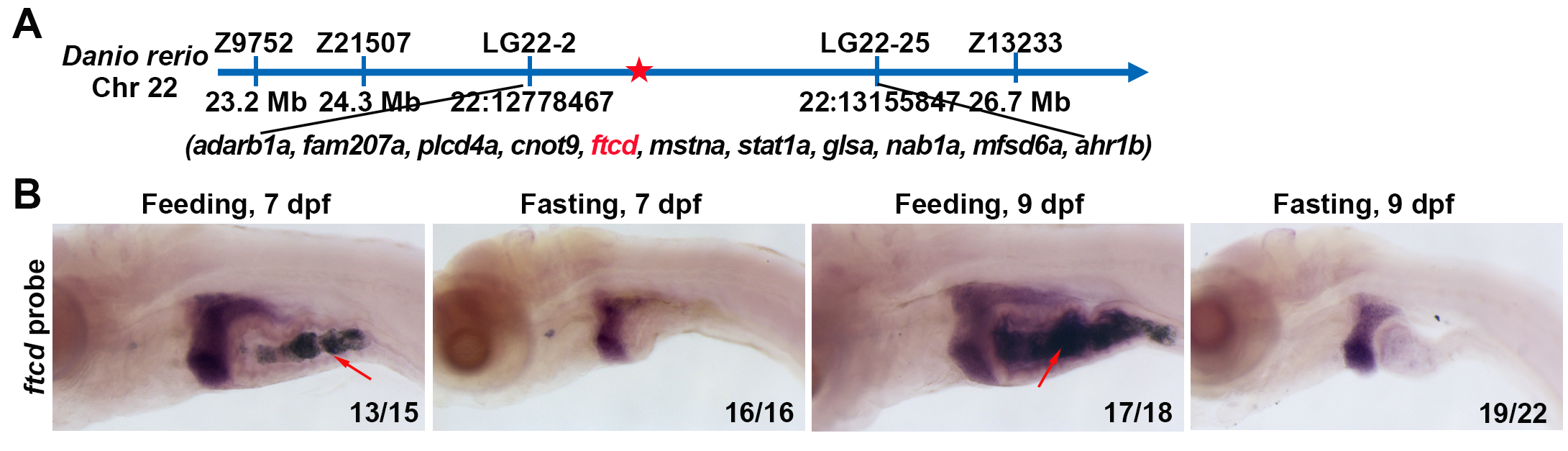

Supplement: S4 Fig — (A) Position cloning narrows down the mutated gene of lvh to chromosome 22 (blue line) in a region including the ftcd locus. (B) WISH images showing the expression pattern of ftcd at 7 dpf and 9 dpf under both feeding and fasting. The red arrowheads indicate food in the gut. (TIF) [file pgen.1009980.s004.tif]

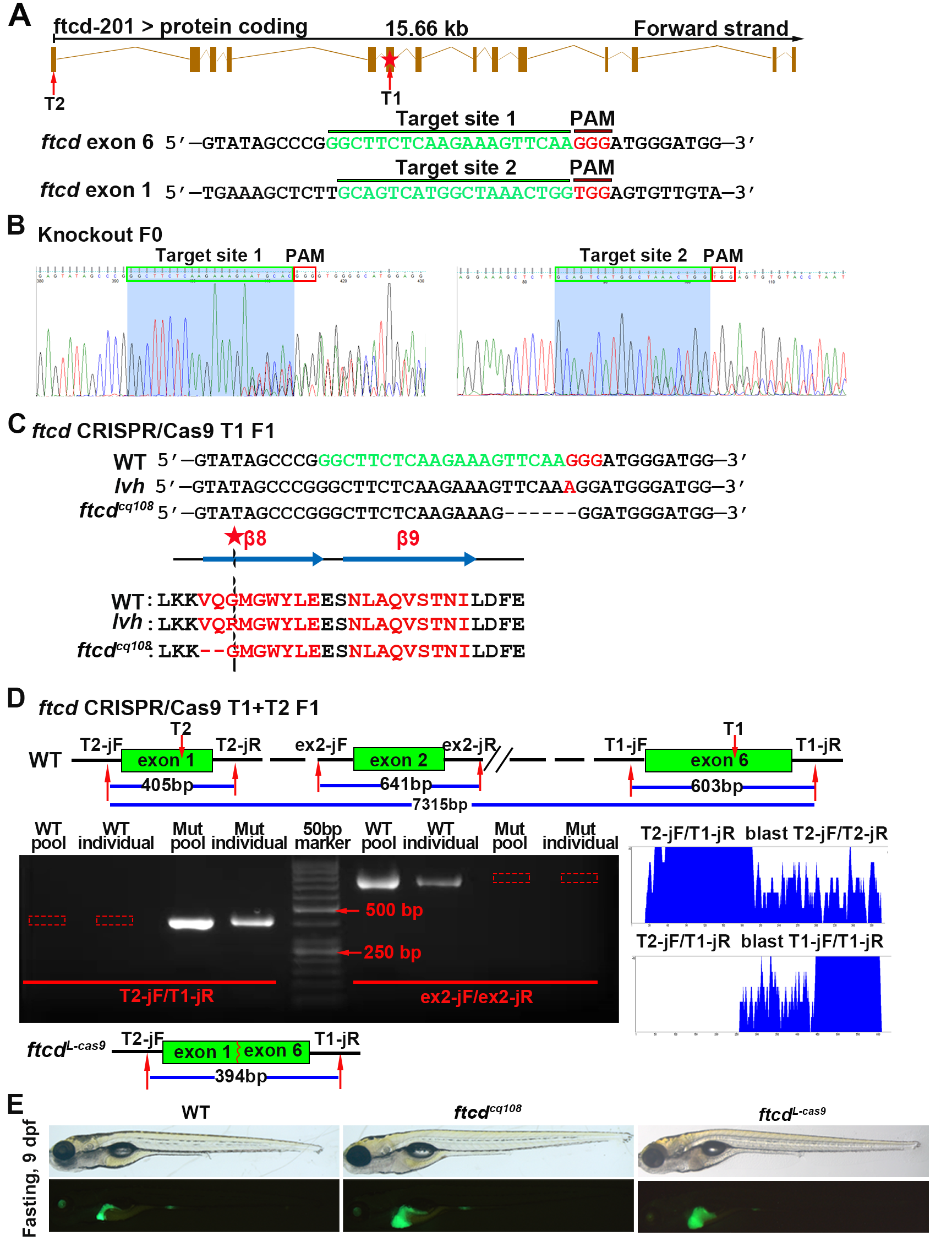

Supplement: S5 Fig — (A) The schematic illustrations of ftcd mutant generation using the CRISPR/Cas9 system. The sgRNA target and PAM region are displayed in green and red, respectively. (B) The two Cas9 target sites for knockout ftcd gene worked in knock out F0 generation. (C) A ftcd Cas9 mutant line by the single-target site generation and the FTCD protein sequences of wild-type, lvh, and ftcdcq108 at the β8 motif region. (D) A large fragment knockout Cas9 mutant of ftcd by two target sites generation. (E) Bright-field and fluorescent images showing the hepatomegaly phenotype of ftcdcq108 mutant and ftcdL-ca9, with the body shape unaffected. WT, wild-type. (TIF) [file pgen.1009980.s005.tif]

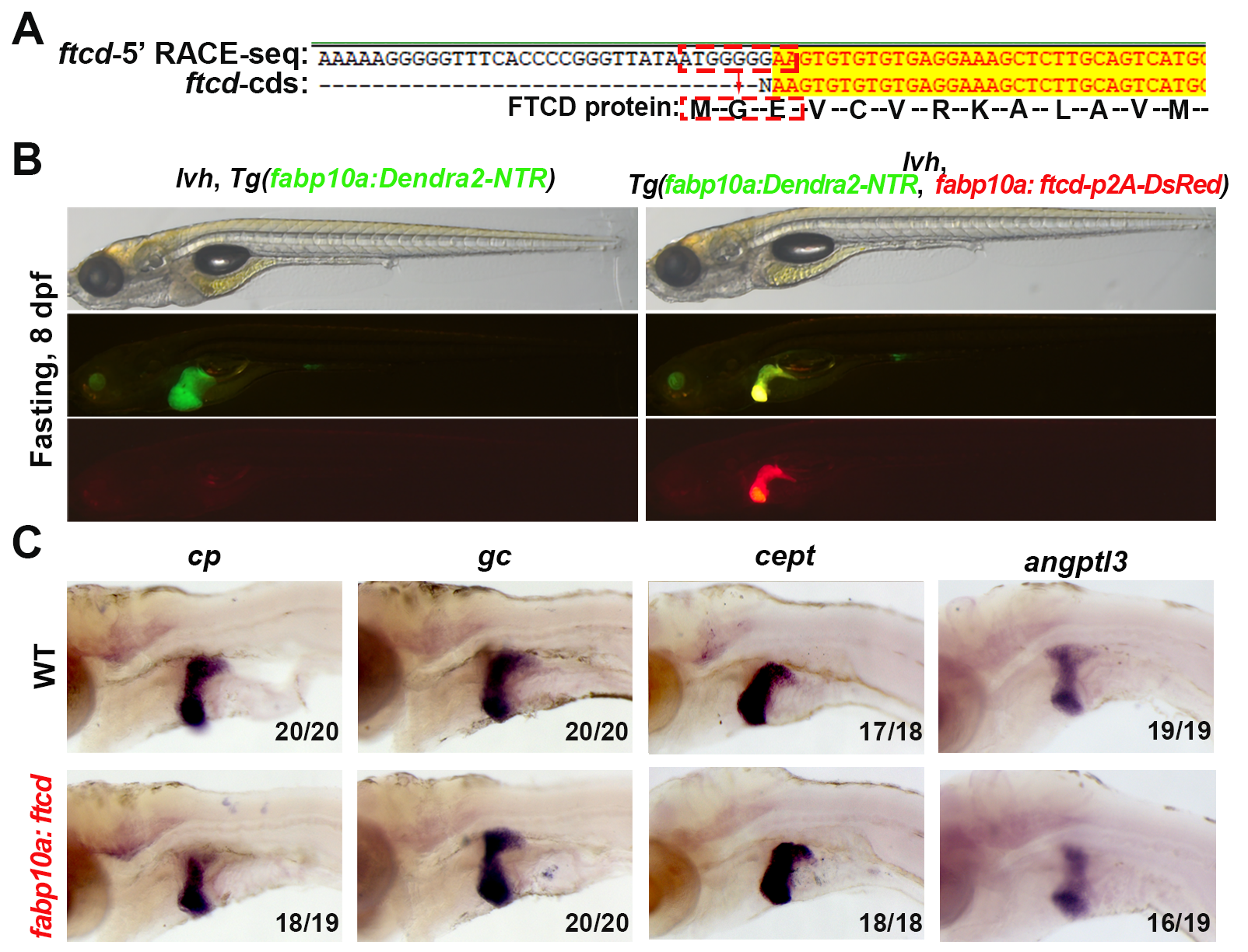

Supplement: S6 Fig — (A) Obtain of ftcd full-length CDS sequence by 5’-RACE. (B) Bright-field and fluorescent images of lvh with or without ftcd overexpression.(C) WISH images showing the expressions of cp, gc, cept, and angptl3 in WT with or without ftcd overexpression. WT, wild-type. (TIF) [file pgen.1009980.s006.tif]

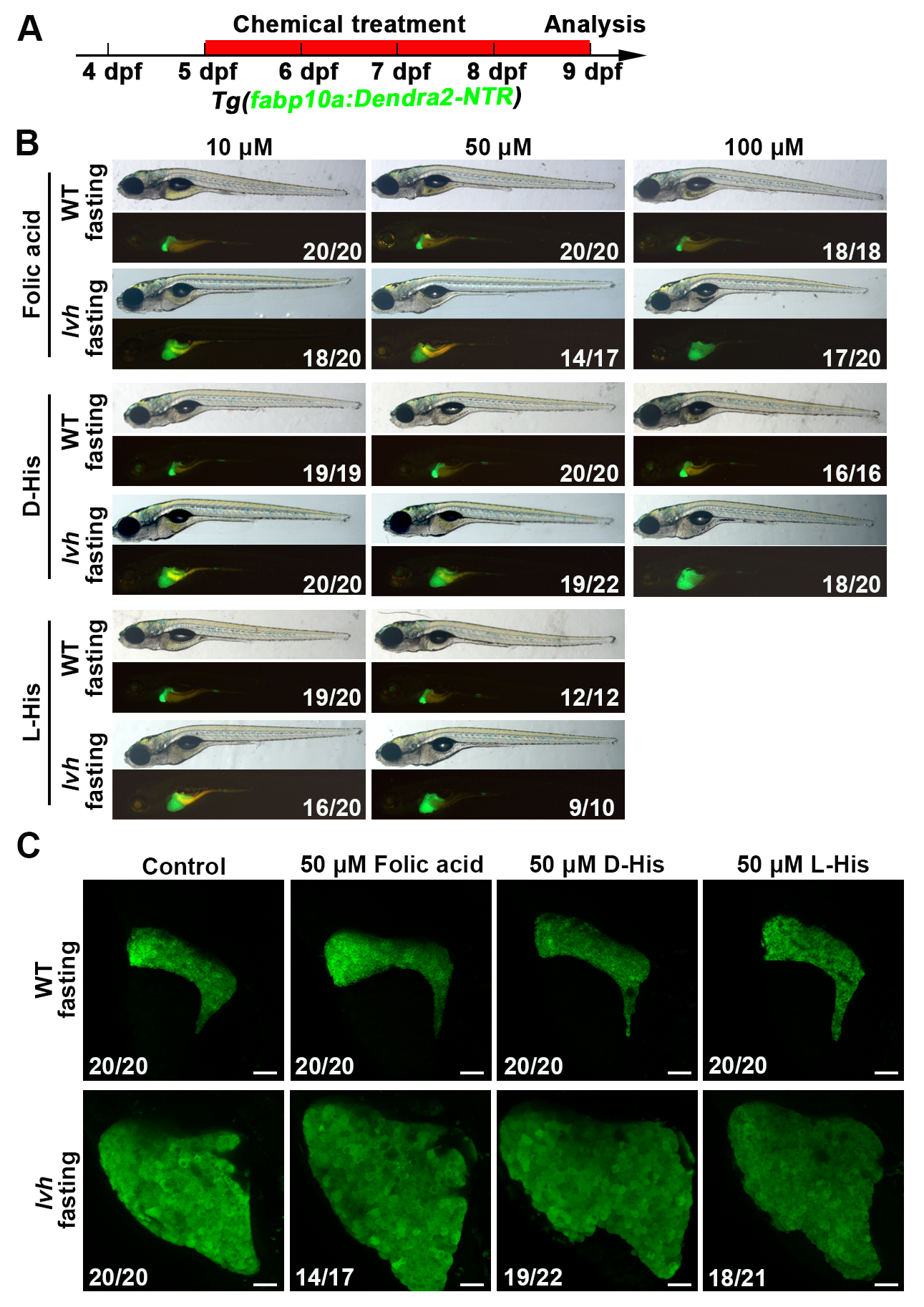

Supplement: S7 Fig — (A) Experimental scheme illustrating the stage of chemicals treatment to wt and lvh. (B) Bright-field and fluorescent images showing the liver morphology of wt and lvh were treated with folic acid, D-His or L-His. (C) 3D confocal projection images showing the liver morphology of wt and lvh were treated with folic acid, D-His, or L-His at fasting 9 dpf. WT, wild-type. Scale bars, 50 μm. (TIF) [file pgen.1009980.s007.tif]

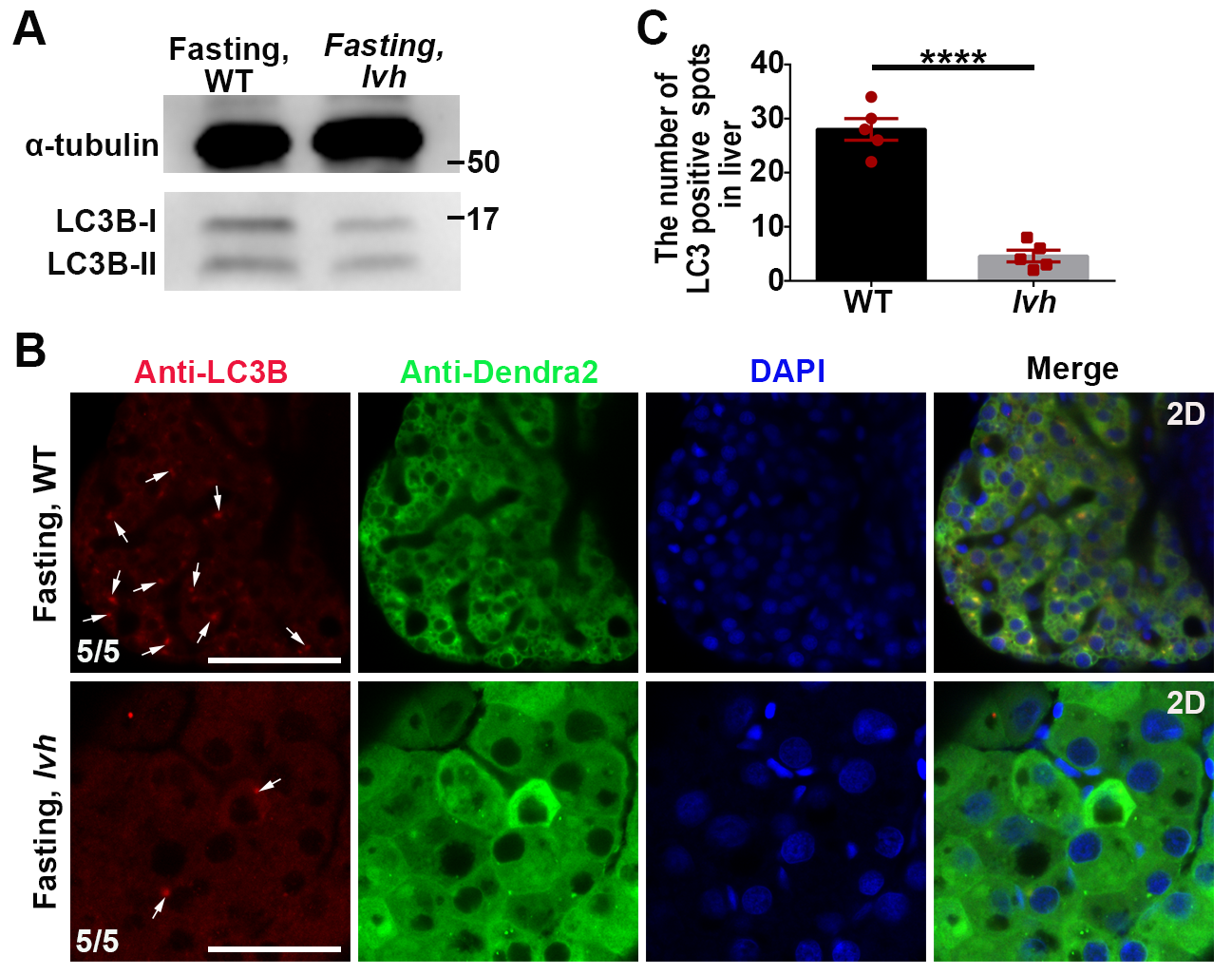

Supplement: S8 Fig — (A) Western blot analysis of LC3B using wt and lvh liver lysates (n = 120). (B) Immunostaining for LC3B and Dendra2 in livers of wt and lvh at fasting 9 dpf (2D imaging). Nuclei were stained with DAPI (blue). (C) Unpaired Student’s t-test for the number of LC3B positive spots in the wild-type (n = 5) and lvh (n = 5). WT, wild-type. Scale bars, 50 μm. (TIF) [file pgen.1009980.s008.tif]

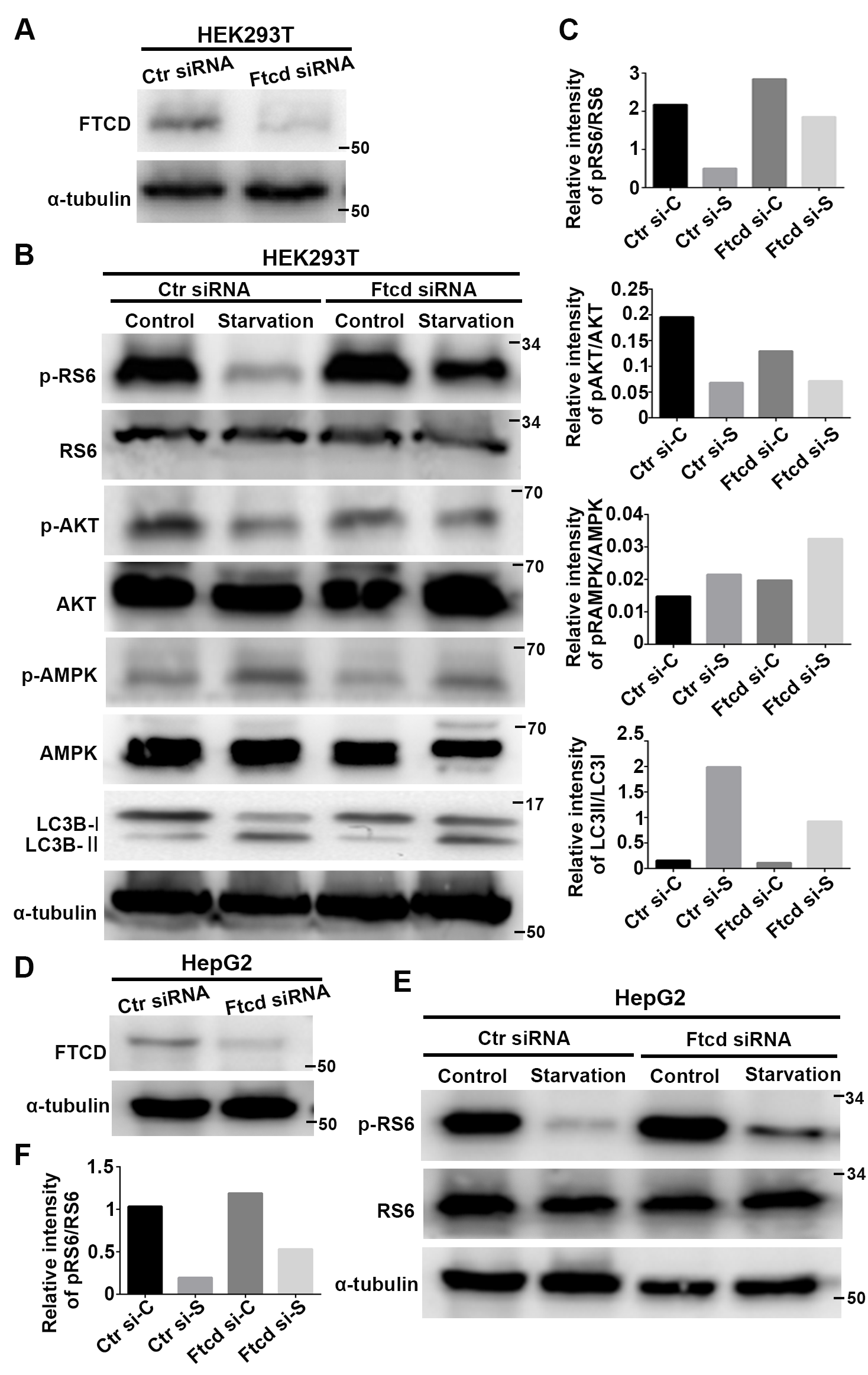

Supplement: S9 Fig — (A) In HEK293T cells, knockdown of FTCD by siRNA duplexes. After transfection for 48 h, the cells were analyzed by Western blotting with antibodies to FTCD and a-tubulin. (B) Western blot analysis of p-RS6, RS6, P-AKT, AKT, P-AMPK, AMPK, and LC3B use HEK293T cells transfected with control siRNA or FTCD siRNA and without starvation (Control) or starved for 4 h. (C) The relative band intensity of the Western blot in B. (D) In HepG2 cells, knockdown of FTCD by siRNA duplexes. After transfection with 48 h, the cells were analyzed for Western blotting with antibodies to FTCD and a-tubulin. (E) Western blot analysis the expressions of p-RS6 and RS6 using HepG2 cells transfected with control siRNA or FTCD siRNA under starvation (4 h) and control. (F) Relative band intensity of p-RS6/RS6 in E. (TIF) [file pgen.1009980.s009.tif]
