# Supplementary material for: Formimidoyltransferase cyclodeaminase prevents the starvation-induced liver hepatomegaly and dysfunction through downregulating mTORC1
Source: PLoS Genet. 2021 Dec 23;17(12):e1009980. doi: 10.1371/journal.pgen.1009980 (PMC8741050; doi:10.1371/journal.pgen.1009980)
Supplement: S1 Table — (DOCX) [file pgen.1009980.s010.docx]

| **A. Sequences of primers used for *in situ* probe synthesis in WISH.** | | |
| --- | --- | --- |
| **Name** | **Primer** | **Nucleotide Sequence 5’-3’** |
| *cp* | forward | GATCTGAGACAGACATCCAC |
| *cp* | reverse | attgtaatacgactcactatagggCAGGTTGCCGAAGACTAAAC |
| *gc* | forward | GCTTTAATAGTCCCAGCATTGC |
| *gc* | reverse | attgtaatacgactcactatagggCCTTAAGCAGCACTGTCATC |
| *ftcd* | forward | GGAAAGCTCTTGCAGTCATGG |
| *ftcd* | reverse | attgtaatacgactcactatagggTCTACAGATCTCTTCATACACC |
| *fabp2* | forward | TCAGGACTTGAAATCTCCGC |
| *fabp2* | reverse | attgtaatacgactcactatagggTCCAACATGAATCACGACACG |
| *ins* | forward | GGTCGTGTCCAGTGTAAGCA |
| *ins* | reverse | attgtaatacgactcactatagggCAGGTGTTTCTGGCATCGG |
| *Trypsin( prss1)* | forward | TCAACAGCTTCACCATGAAGG |
| *Trypsin(prss1)* | reverse | attgtaatacgactcactatagggTGCCAGATGGTATTGCAACAC |
| *apoa2* | forward | GCAGACTCAGCTGGTGCAGT |
| *apoa2* | reverse | attgtaatacgactcactatagggGCTGGACGGGATGTTATTTTC |
| *cetp* | forward | TCGCTCTGGTCAAGATAAGC |
| *cetp* | reverse | attgtaatacgactcactatagggTGGCTGTAGACAGCTCAGTC |
| *mttp* | forward | TGGTGGATGCAGTAACATCAG |
| *mttp* | reverse | attgtaatacgactcactatagggTGCGATCAGAGACTCCAGAC |
| *angptl3* | forward | TCAACGACATCTTCCAGAAG |
| *angptl3* | reverse | attgtaatacgactcactatagggTCCAACGTGAAGGTGTACTCG |
| *cpt1aa* | forward | TGGCAGAAGCTCATCAGGCT |
| *cpt1aa* | reverse | attgtaatacgactcactatagggTGCTGTCAGACACGTGCTGT |
| *acsl2* | forward | TGGCTGAGATCTCGATGGTG |
| *acsl2* | reverse | attgtaatacgactcactatagggGGTAATGGAGCACCAACATG |
| *mgll* | forward | GGTGTTCCTTACAGCGACCT |
| *mgll* | reverse | attgtaatacgactcactatagggTCCAGGATCCAGGTGCTCAC |
| *lpl* | forward | TGACGGCTCTGTATGATCGTG |
| *lpl* | reverse | attgtaatacgactcactatagggTGATCCTCATTCTGCGGATGG |
| **Underlined are T7 primer sequences.** | | |
| **B. Sequences of primers used for plasmid generation and gene detection.** | | |
| **Name** | **Primer** | **Nucleotide Sequence 5’-3’** |
| *Ftcd-full-cds* | forward（+*BamH*1） | cgggatccTTATAATGGGGGAAGTGTGTG |
| *Ftcd-full-cds* | reverse（+HA tag） | tgtacccgattatgccggaagcTCATTTCCTCTGGTCTGCCG |
| *HA-P2A-DsRed* | forward | TGAAGCAGGCTGGAGATGTGGAGGAGAACCCTGGACCTATGGCCTCCTCCGAGAACGTC |
| *HA-P2A-DsRed* | reverse | CTACAGGAACAGGTGGTGGC |
| *Ftcd-exon1* | forward | TGACCTGAATCCAGTCCAAGA |
| *Ftcd-exon1* | reverse | TGCTGTTTACACAGTGACAAT |
| *Ftcd-exon2* | forward | TCCAGGTTATTGACGCCATAG |
| *Ftcd-exon2* | reverse | TGACCTTATCTGGTAATGCC |
| *Ftcd-exon6* | forward | TGAGGCCTAATTTCTGAGGA |
| *Ftcd-exon6* | reverse | TGAGACTGATTCCTGATATGC |
| *Ftcd-gRNA-T1* | forward | taatacgactcactatagGGCTTCTCAAGAAAGTTCAAgttttagagctagaaatagca |
| *Ftcd-gRNA-T2* | forward | taatacgactcactatagGGCAGTCATGGCTAAACTGGgttttagagctagaaatagc |
| *gRNA* | reverse | AAAAAAAGCACCGACTCGGTGCCAC |
| **C. Sequences of primers used for qPCR** | | |
| **Name** | **Primer** | **Nucleotide Sequence 5’-3’** |
| *Z-actb2* | forward | GTGCGGAATATCATCTGCTTG |
| *Z-actb2* | reverse | TGCGGCAATTTCATCATCCAT |
| *cp* | forward | CAGCACACTTCCACGGCCAC |
| *cp* | reverse | GGTCGGTCACATGACAGTGC |
| *gc* | forward | GCTCGCAGTACTCCAAACTGG |
| *gc* | reverse | ACAGTTGTGCCGGTGAGAGC |
| *cpt1aa* | forward | CATCCTTAGGCCTGCTCTTCAAA |
| *cpt1aa* | reverse | ACCATGACACCCCCAACTAACAT |
| *cpt1ab* | forward | TTGTCCACCAGTCAGACTCC |
| *cpt1ab* | reverse | ATGGCTTGAGTGTTTGCTGG |
| *acs1b* | forward | GACGAGTTCGGTCGAGTTCA |
| *acs1b* | reverse | TGCGCAGATAATCCCTCACC |
| *acsl2* | forward | TGGTCACTAGACCGAGACCAAT |
| *acsl2* | reverse | TACGCTGTCTTTGTGTCCTC |
| *mgl* | forward | CGAGAGGCCGCAGGATTTTA |
| *mgll* | reverse | TGAGTTTAGGAGCCAAGCG |
| *lpl* | forward | TTGGCGCTCATGTTGCAGG |
| *lpl* | reverse | TGGAGAACCACGGGTGTTG |
| *lipca* | forward | CACTGGCAAAAGCAAGAGGC |
| *lipca* | reverse | TCTCCTCGACAAGTGTTATGGG |
| *lipcb* | forward | TGGCTCATGTGGACTTCTACC |
| *lipcb* | reverse | GCGAATCAGTGAACAGTCTGA |
| *apoa2* | forward | ATGAAGCTGACATTCGCTCTC |
| *apoa2* | reverse | TAGTGCTGGCTCAACTGCAG |
| *cetp* | forward | GTGCTGTTCCTGGTTGTCGC |
| *cetp* | reverse | TCCGTATTTGACCGTCCCAA |
| *mttp* | forward | TGGAGTTCAGCTTGTGGTACA |
| *mttp* | reverse | CTCTGAGAACTGGACGGTGGTG |
| *angptl3* | forward | TGAACATGGATTTGGGAAAC |
| *angptl3* | reverse | TCCAACGTGAAGGTGTACTCG |
